# Franchising structure changes and shareholder value: Evidence from store buybacks and refranchising

**DOI:** 10.1007/s11747-022-00921-3

**Published:** 2023-01-14

**Authors:** Anna Sadovnikova, Manish Kacker, Saurabh Mishra

**Affiliations:** 1 Leon Hess Business School, Monmouth University, 400 Cedar Avenue, West Long Branch, NJ 07764 USA; 2 DeGroote School of Business, McMaster University, 1280 Main Street West, Hamilton, ON L8S 4M4 Canada; 3 School of Business, George Mason University, 4400 University Drive, Fairfax, VA 22030 USA

**Keywords:** Franchising, Agency theory, Transaction cost analysis, Marketing-finance interface, Event study

## Abstract

Drawing on agency theory and transaction cost analysis, this study investigates the impact of refranchising and buybacks of downstream retail units by franchising firms on shareholder value (i.e., stock returns). It further evaluates the contingency role of firm and industry factors in shaping this impact. An event study analysis over the years 2001–2020 confirms that both refranchising and buybacks positively affect stock returns. However, notable impact differences emerge between the two types of strategic decisions. For refranchising, firms with lower royalty rates, smaller returns-on-assets (ROA), and higher trade credit provided generate higher stock returns. Whereas, for buybacks, firms with higher royalty rates derive more value in stock markets. Analysis further shows that investors judge refranchising (buybacks) less (more) favorably in munificent industries, but industry dynamism has no effect on the stock returns generated from these moves. Together, the study offers important implications for franchising theory and retail practice in marketing.

**Supplementary Information:**

The online version contains supplementary material available at 10.1007/s11747-022-00921-3.

Franchising is an important distribution strategy for firms across industries. In the United States alone, franchising has contributed $787.7 billion in economic output and employed close to 8.2 million people in 2021 (Niu, 2022). In response to evolving market conditions, firms operating with franchise systems must periodically make strategic decisions regarding structure of their downstream retail channels (e.g., Hsu et al., 2017). In particular, recognizing that a well-designed franchising system is a driver of competitive advantage (e.g., Palmatier et al., 2020), managers have to decide whether to decrease or increase the proportion of company-owned to franchised units through refranchising or buybacks^1^ of retail stores (Srinivasan, 2006). For example, in the past few years, McDonald’s has announced increases in the share of franchised units by refranchising several of its company-owned and operated restaurants (Forbes, 2018), while Applebee’s has announced buybacks of some of its restaurants from franchisees to operate as company-operated units (Restaurant Business, 2018).

Although firms use franchising as an organizational form in retailing, the shareholder implications of discrete strategic moves that alter the degree of reliance on franchisees in retail channels have received limited attention from marketing scholars. Notably, some scholars have articulated the managerial benefits of having the right mix of franchised and company-owned retail units (e.g., Lafontaine & Kaufmann, 1994) and others have linked strategy relying on both franchisee and company-owned downstream retail units to financial metrics of firms (e.g., Srinivasan, 2006). Further, recent work has documented the financial implications of changes to franchising structure for firms (Hsu et al., 2017). Despite the important contributions provided by these studies, investigations disentangling the shareholder effects of the two types of franchising structure change decisions (i.e., refranchising and buybacks) that determine the proportion of franchising to company owned retail units, and the boundary conditions that differentially bear upon these shareholder effects, remain to be conducted (see Table 1). Given the financial and strategic significance of franchising systems, we submit that this is an important gap and take a step to bridge it in this study (see Fig. 1). Specifically, we ask the following questions:

- Do refranchising and buyback announcements of existing downstream retail units by franchising firms affect their shareholder value?
- What moderating influence do firm and industry level factors have on these focal effects?

**Table 1:** Studies in marketing on performance impact of refranchising and buybacks

Author(s)	Key Question	Evaluate Refranchising & Buybacks Separately	Contingency Factors		Financial Market-Based Performance Impact	Abnormal Stock Returns	Event-Specific Abnormal Stock Returns
			Firm Level	Industry Level			
Dahlstrom and Nygaard (1994)	Examine and explain differences in location and sales revenues between franchised and franchisor-operated outlets	No	Yes	No	No	No	No
Kalnins (2004)	Comparison of impact of addition of new units on revenues per room of geographically proximate incumbent units for franchised and non-franchised hotel chains in Texas	No	Yes	No	No	No	No
Butt et al. (2018)	Measure how the governance of a retail outlet (franchised or franchisor-operated) impacts sales performance of the outlet when clustered with other outlets of the same brand	No	Yes	No	No	No	No
Srinivasan (2006)	Measure the relationship of a firm’s dual distribution strategy with firm value (as measured by Tobin’s Q), both individually and in the context of firm characteristics	No	Yes	No	Yes	No	No
Madanoglu et al. (2011)	Comparison of risk-adjusted financial performance of franchising vs. non-franchising restaurant firms in the US	No	No	No	Yes	Yes	No
Hsu et al. (2017)	Understand the relationship between franchise ownership structure and firm stock market performance using panel data methods. Document the moderating role of firm strategic investment emphasis between tangible & intangible assets	No	Yes	No	Yes	Yes	No
*This Paper*	*Evaluate the effects of Refranchising and Buybacks on firm abnormal stock returns and highlight firm and industry level contingencies*	*Yes*	*Yes*	*Yes*	*Yes*	*Yes*	*Yes*

**Fig. 1:**
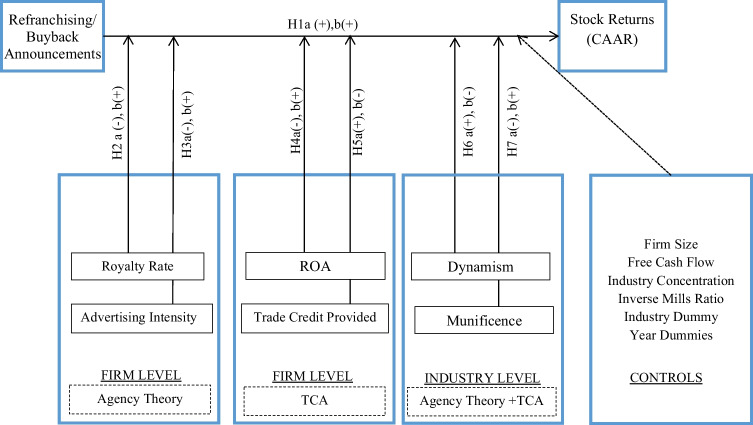
Theoretical framework

To inform our inquiry, we theoretically draw on agency theory and transaction cost analysis (TCA)—two key pillars of the efficient contracting perspective on the organization of economic activity (Combs & Ketchen, 1999; Mahoney, 1992; Williamson, 1985) (see Fig. 1). Our approach is in line with extant literature, which has called upon agency theory (e.g., Lafontaine, 1992) and TCA (e.g., Minkler & Park, 1994) to understand franchising (Combs et al., 2011; Dnes, 1996). In particular, deriving from these theories, we illustrate how refranchising and buybacks entail advantages and disadvantages for firms, which can affect prospective cash flows to reflect in the firms’ shareholder value (e.g., Srivastava et al., 1998).

While evaluating the shareholder effects of refranchising and buybacks, we recognize the importance of evaluating contingency factors influencing these effects (e.g., Hsu et al., 2017; Srinivasan, 2006). Agency theory underscores information asymmetry, incentive misalignment, and environmental uncertainty as three major forces governing principal-agent relationships, such as those between franchising firms and franchisees (e.g., Bergen et al., 1992). Similarly, TCA highlights the behavioral and environmental uncertainties associated with working with channel partners (such as franchisees) and company owned units, which can adversely affect firm performance (e.g., Rindfleisch & Heide, 1997). Building on these observations, we evaluate royalty rate, advertising intensity, returns-on-assets (ROA), and trade credit provided by franchising firms as firm-level moderators in our framework.

Royalty rate and advertising intensity reflect the quality of resources of franchising firms and the emphasis placed by the firms on appropriating value generated from the resources (Combs & Ketchen, 2003; Mizik & Jacobson, 2003). Agency theory indicates that these factors are likely to affect the incentive misalignment between channel partners and expose franchising firms to moral hazard and free riding by franchisees (Combs & Ketchen, 2003; Lafontaine, 1992; Michael, 1999), moderating the shareholder returns from franchising structure changes made by the firms. In contrast, ROA and trade credit provided reflect the ability of firms to utilize their current assets (Homburg et al., 2014; Srinivasan, 2006) and the quality of their relationships with channel partners (e.g., Astvansh & Jindal, 2022; Frennea et al., 2019), respectively. Based on TCA, these factors can regulate some of the behavioral uncertainties associated with franchisees, affecting stock returns from franchising decisions made by firms.

Additionally, previous studies analyzing abnormal stock returns to channel related announcements have underscored the importance of looking at boundary conditions across both firm and industry levels (e.g., Geyskens et al., 2002; Homburg et al., 2014). Therefore, we also consider the moderating effects of two industry-level factors (e.g., Feng et al., 2017) – industry munificence and dynamism – in our study. Our focus on these factors is again guided by agency theory and TCA, which detail the role of environmental uncertainty in shaping agency and transaction costs of working with channel partners and internal employees.

We assess the hypothesized relationships with data collected from multiple archival sources of information. A sample of 205 announcements (with 125 refranchising and 80 buyback announcements) made by publicly traded firms across multiple industries in the United States over the years 2001–2020 provide the empirical context for our analysis. We employ the event study methodology to estimate the effects of refranchising and buyback announcements on abnormal stock returns of firms. This methodology captures stock market impact of unexpected announcements made by firms, while minimizing endogeneity concerns (Sorescu et al., 2017). Further, we recognize that refranchising and buybacks constitute opposing franchising strategies in terms of governance structures. As such, we follow previous research in marketing for analyzing strategic decisions that reflect opposite strategic pathways for firms (Wiles et al., 2012), to separately analyze moderating effects of firm and industry level factors on the stock market consequences of refranchising and buyback announcements.

Our results confirm that both refranchising and buyback announcements by firms enhance shareholder value. Further, an examination of firm level factors reveals that firms that have lower royalty rates, earn lower ROA, and provide higher trade credit to downstream channel partners derive more value in the stock markets from refranchising. On the other hand, firms with higher royalty rates are observed to earn greater value for shareholders from buybacks. Our results also show that refranchising/buybacks create less/more shareholder value in munificent industries; while, industry dynamism is observed to have no effect.

Our work contributes to both marketing theory and retailing practice in multiple ways. We are the first to document that changes in the franchising system through *both* refranchising and buybacks of downstream retail units create value for shareholders in the financial markets (see Table 1). There has been an intense debate among scholars regarding the extent to which retail firms should rely on franchising over time (e.g., Lafontaine & Kaufmann, 1994). Some have maintained that franchisors benefit from increasing the proportion of company owned and operated outlets over time (e.g., Dant & Kaufmann, 2003; Oxenfeldt & Kelly, 1968). In contrast, others have pointed to the synergistic effects of having both company and franchisee owned retail outlets for firms (e.g., Bradach, 1997; Srinivasan, 2006), and have investigated if there is a steady state level of franchising for retail firms (Lafontaine & Shaw, 2005). We take the efficient contracting perspective to theoretically illustrate the advantages and disadvantages of refranchising and buybacks, two seemingly opposite distribution strategies, for franchising firms and confirm that financial markets reward firms for both decisions. As such, our findings underscore that no one form of franchise system is superior, and markets reward firms that are willing to make efficiency-driven adjustments to their distribution structures.

Furthermore, the few studies that have examined franchising strategy and firm value (Hsu et al., 2017; Srinivasan, 2006), have not separated buybacks and refranchising (see Table 1). Doing so allows us to offer a nuanced perspective on (and an enhanced understanding of) the firm and industry level conditions affecting the franchising levels/shareholder value relationship. Specifically, managers of franchising firms with high royalty rates and those operating in munificent industries can infer that they are likely to generate lower (higher) stock returns from refranchising (buybacks). Further, managers who are already delivering high ROA are advised to take their refranchising decisions with more deliberation, as they are likely to create lower stock returns from such moves. Finally, firms investing more in downstream relationships, as reflected in higher trade credit provided by them to partners, are likely to benefit shareholders more if they refranchise. To our knowledge, we are the first to offer these insights across buybacks and refranchising, providing theory and practice implications not available in extant marketing strategy research in general, and franchising research in particular.

## Conceptual framework

In evaluating the financial consequences of franchising, scholars have drawn on two key pillars of the efficient contracting perspective—agency theory and transaction cost analysis (TCA) (e.g., Dahlstrom & Nygaard, 1999; Hsu et al., 2017).^2^ Both agency theory and TCA illuminate the downsides and benefits of different governance structures for firms. Since franchising decisions (i.e., refranchising and buybacks) change the extent of hierarchical vs. market-based governance utilized by firms in their retail channels, the two theories are useful in guiding which franchising structures are more efficient for firms and under what conditions.

Agency theory recognizes post-contractual problems for firms (i.e., principals) in working with agents (e.g., Bergen et al., 1992). Specifically, Bergen et al. (1992; p. 3–4) identify three elements of agency theory that can influence the performance of principals. First, agents are driven by self-interest and that this incentive misalignment can induce them to work towards maximizing their own welfare, with limited regard to interests of the principals. This assumption underscores the risk of moral hazard (with agents potentially freeriding on the effort and resources of principals), which can detract from the principals’ performance. Second, there is information asymmetry between principals and agents, which exacerbates moral hazard by affording self-interested agents the possibility to shirk their responsibilities and hurt the principals. Third, environmental uncertainty makes it difficult for principals to effectively govern their relationships with agents.

TCA aligns with agency theory in underscoring the different behavioral and environmental uncertainties faced by firms when making transactions (Rindfleisch & Heide, 1997; Williamson, 1985). In particular, TCA highlights that firms face governance problems related to (a) uncertain behaviors of partners and (b) uncertainties induced by the environment in which firms operate (Rindfleisch & Heide, 1997; p. 46). TCA then points out that, to deal with these governance issues, firms need to implement communication and coordination efforts with partners and find ways to adjust to changes in the environment, with all these efforts entailing transaction costs that detract from firm performance (Rindfleisch & Heide, 1997).

Although agency theory and TCA highlight downsides, they also underscore gains that accrue to firms from dealing with outside partners and internal employees (which we list in detail below in arguing the hypotheses). Together, the two theories provide a useful lens through which to evaluate the shareholder value (i.e., stock returns) consequences of refranchising and buyback decisions of firms.

### Shareholder value of refranchising and buybacks

Regarding the advantages of franchising, agency theory and TCA highlight efficiency-related benefits that accrue to franchisors from the unique skills, competencies, outlet specific know-how, and local market knowledge of the franchisees (e.g., Heide, 1994; Windsperger & Dant, 2006). Together, these benefits add to the internal knowledge base of franchisors (Sorenson & Sørenson, 2001) and can help them to innovate (Bradach, 1997). Additionally, franchising provides strategic options to managers, where they can terminate existing partnerships and align with new franchisees based on changing market conditions (Balakrishnan & Wernerfelt, 1986)—often at lower transactional costs than if they attempted to manage these changes within the boundaries of their firms. Such strategic options can be a source of competitive advantage, as they allow franchising firms to better manage distributional challenges and nimbly respond to market needs (Palmatier et al., 2020). Together, these observations suggest that refranchising can enhance future cash flows of franchisors, adding to their shareholder value.

Despite these benefits, firms can also face significant headwinds with greater reliance on franchisees. Specifically, agency theory alerts that franchisors face the risks of franchisees unfairly exploiting their assets such as brands and business format expertise due to information asymmetries and incentive misalignment (Bergen et al., 1992; Combs et al., 2011). To protect against the possibility of unfair exploitation of franchisor market-based assets (and the resulting reduction of future cash flows) due to horizontal and/or vertical free riding by franchisees (Mathewson & Winter, 1985), franchisors need to incur the coordination costs of monitoring franchisees on an ongoing basis (e.g., Agrawal & Lal, 1995). Aligned with this, TCA indicates that to manage the behavioral uncertainties associated with franchisees, franchisors would need to undertake coordination and communication efforts that can increase their transaction costs (e.g., Rindfleisch & Heide, 1997), reducing their future cash flows and lowering shareholder value.

One solution to these problems can be to realize the coordination benefits of fiat from an increased reliance on hierarchical governance (Williamson, 1985) through buybacks of downstream retail units. Greater reliance on company-operated stores allows franchisors to increase bargaining power to manage franchisees more cost effectively (Bradach, 1997; Michael, 2000). It also enables franchisors to have direct interaction with customers at a larger number of locations. This should lead to superior customer knowledge for firms, allowing them to offer better customer experiences to positively affect financial performance. However, it is also worth noting that store buybacks can be expensive, placing downward pressures on future cash flows of firms. Additionally, agency theory argues that franchisors can have agency issues with internal agents, i.e., managers of company owned stores, with these agents not performing their roles adequately (e.g., Brickley & Dark, 1987; Norton, 1988; Rubin, 1978). This can necessitate firms to place efforts in monitoring employees and providing higher performance-based incentives to them, which can dampen their prospective cash flows.

In summary, there are arguments both in favor and against whether firms should have higher or lower levels of franchising in the retail chain. We contend that, ultimately, the right level of franchising for firms would be where distribution channel governance structures are appropriately aligned with agency issues and transactional costs dimensions. The “Darwinian economics” rationale advanced by Anderson (1988) holds that competitive market forces compel firms to select strategies that approximate optimal behavior. Indeed, previous research provides evidence that financial markets are supportive of the Darwinian rationale by showing that investors reward firms for both brand acquisitions and brand disposals based on the context (Wiles et al., 2012). With respect to retail channels, firms would similarly benefit from adjusting their franchising levels at discrete intervals through refranchising or buybacks based on their unique conditions. Since investors understand that distribution channels are market-based assets with financial value (Srivastava et al., 1998) and that firms adjust channel structures infrequently, they would likely reward firms for both these franchising level changes. Together, we posit:

#### H1a

Announcements of refranchising have a positive effect on stock returns of franchising firms.

#### H1b

Announcements of buybacks have a positive effect on stock returns of franchising firms

### Forces governing shareholder value of refranchising and buybacks

Following previous research, we contend that factors across firm and industry levels would present boundary conditions (e.g., Geyskens et al., 2002) for the impact of refranchising and buybacks on stock returns. With respect to firm-level factors, we first derive from agency theory to focus on royalty rate and advertising intensity of franchising firms as firm level moderators. Next, using the TCA lens, we outline the role of ROA and trade credit provided in shaping the stock returns to franchising structure change announcements made by firms.

#### Firm-level factor: Royalty rate

Extant literature suggests that the royalty rate paid by franchisees reflects the brand value of the franchisors and the quality of inputs and services provided by them to the franchisees (Combs & Ketchen, 2003; Lafontaine, 1992). Agency theory would predict that franchisors that provide access to valuable brands and offer higher value-added services to their retail units are likely to face greater risks of free-riding and moral hazard by franchisees (e.g., Norton, 1988; Rubin, 1978). As brands are key strategic resources for firms, any actions that dilute them are likely to lower future cash flows of the firms (e.g., Wiles et al., 2012). Additionally, inputs and services offered to franchisees entail costs and if franchisees do not perform as expected, the franchising firms face the risks of not recouping these investments. Franchisors can mitigate some of these agency concerns through more stringent monitoring of franchisees (Mathewson & Winter, 1985). However, such monitoring can be expensive and take away scarce resources from other productive uses. Together, these observations suggest that when firms make refranchising announcements, the cash flow gains are likely to be lower if firms are also charging higher royalty rates, which would reflect in reduced stock returns.

With respect to buybacks, theory would predict the opposite effect on franchising firms’ stock prices. As previously noted, higher royalty rates reflect greater levels and quality of ongoing services provided by franchisor to franchisees (Norton, 1988; Rubin, 1978). In business contexts that require close coordination between franchisor inputs and franchisee efforts, Muris et al. (1992) have empirically shown that ownership of downstream retail units presents a superior organizational form. Additionally, Michael (2002) has argued that franchised chains (relative to company owned chains) are less able to coordinate different elements of marketing strategy. To the extent that a higher level of franchisor inputs and brands (as reflected in higher royalty rates) suggest a need for greater coordination for effective implementation of a franchising firm’s distribution strategy, this indicates that investors would reward firms more when they announce buybacks of retail units and have higher royalty rates. Together, we posit:

##### H2a

The positive effect of refranchising on stock returns of franchising firms is lower when the royalty rate charged by them to franchisees is higher.

##### H2b

The positive effect of buybacks on stock returns of franchising firms is higher when the royalty rate charged by them to franchisees is higher.

#### Firm-level factor: Advertising intensity

Agency theory would also indicate that stock returns derived by firms from refranchising would be lower when their advertising intensity is high. Higher advertising intensity reflects that advertising is a central element of a firm’s marketing strategy and that the firm is investing in building customer-based resources, such as brands and customer equity (Mizik & Jacobson, 2003). However, Michael (1999) finds that chains with a higher reliance on franchising tend to underinvest in advertising (with franchisees more likely to free-ride on franchisors’ efforts) relative to chains that have a relatively higher proportion of company-owned units. The sub-optimality of franchisee efforts is likely to diminish the productivity of advertising outlays by franchising firms and dilute the franchisors’ brands and customer relationships, magnifying the agency concerns associated with franchising. As firms would need to mitigate these agency concerns through greater monitoring of franchisees, the costs associated with these efforts would reduce some of the financial gains derived from refranchising.

On the other hand, many of these agency concerns associated with franchising will be attenuated if the franchisors buy back existing franchised retail units. Furthermore, advertising is an important mechanism through which firms appropriate value created in the marketplace (Mizik & Jacobson, 2003). This is because advertising leads to persistent and long-lasting informational effects, which can help firms capture gains in consumer surplus (e.g., Mizik & Jacobson, 2003). As firms with high level of advertising intensity capture a bigger share of consumer surplus, they would potentially generate larger cash flows compared to firms that advertise with less intensity. Buybacks enable firms to take ownership of a larger proportion of their downstream retail units, allowing them to keep a higher share of the value created by advertising for themselves. Earlier, we had argued that one potential downside of buybacks is that these strategic moves can be expensive. As advertising helps capture consumer surplus created by firms and buybacks allow firms to keep these within their boundaries, it would compensate for some of negative effects of buybacks on firm cash flows. Along with the other advantages afforded by buybacks (i.e., lower agency issues), this implies that higher advertising intensity will increase the stock returns from buybacks announcements. Together, we posit:

##### H3a

The positive effect of refranchising on stock returns of franchising firms is lower when the firms have higher advertising intensity.

##### H3b

 The positive effect of buybacks on stock returns of franchising firms is higher when the firms have higher advertising intensity.

#### Firm-level factor: Return on assets (ROA)

TCA suggests that the value derived by firms from franchising depends on the benefits offered by franchisees compared with the transaction costs incurred in governing the behavioral uncertainties associated with them (Dahlstrom & Nygaard, 1999). As we noted previously, franchisees offer valuable resources to franchising firms in the form of outlet specific know-how and local market knowledge (e.g., Heide, 1994; Windsperger & Dant, 2006). Additionally, franchisees can help ease resource constraints faced by the franchising firms as they look to grow their business through existing stores (Combs et al., 2011; Norton, 1988). However, in situations where the franchising firms already have high returns on assets (ROA), some of these benefits lose importance. High ROA indicates that firms are able to use their internal assets efficiently (Zou & Cavusgil, 2002) and manage their core functions at lower costs (e.g., David & Han, 2004). As such, high ROA firms are likely to have better management supervision of employees and face relatively fewer cost pressures when growing their business. Less reliance on outside partners, combined with the transaction costs incurred in monitoring and coordinating franchisees, indicate that firms with high ROA would gain less from refranchising.

In contrast, these observations would predict the opposite effect for buybacks. Additionally, greater access to funds made possible by high ROA, would reduce the cash flow pressures associated with buyback of downstream retail units. Further, firms will also not need to share their financial returns with outside franchisees, helping them appropriate higher cash flow gains for themselves. Overall, we expect these gains afforded by higher ROA to enhance stock returns to firms from buybacks of downstream retail units. Together, we posit:

##### H4a

 The positive effect of refranchising on stock returns of franchising firms is lower when the returns-on-assets (ROA) of the firms are higher.

##### H4b

 The positive effect of buybacks on stock returns of firms is higher when the returns-on-assets (ROA) of the firms are higher.

#### Firm-level factor: Trade credit provided

Firms often provide trade credit to downstream partners in channel relationships (Astvansh & Jindal, 2022). Specifically, in the context of franchising, franchisors at times offer financing to their franchisees (Lafontaine, 1992). Frennea et al. (2019) as well as Astvansh and Jindal (2022) present evidence and insights on how provision of such trade credit enhances shareholder value of firms, as it increases the downstream partners’ dependence on the firms. As such, from a TCA perspective, the provision of trade credit to franchisees is likely to enhance their dependence on the franchising firms, reducing information asymmetry between the two parties and lowering monitoring costs (Petersen & Rajan, 1997). The provision of trade credit to franchisees also reflects the relationship quality between franchising firms and franchisees, indicating higher levels of trust and commitment between them (Frennea et al., 2019). This strengthening of relational norms should reduce the likelihood of franchisee opportunism and safeguard the interests of the franchising firms. Based on TCA, this serves as another reason why trade credit provision by franchising firms should increase the financial attractiveness and value relevance of refranchising for the firms.

In contrast, when franchisors provide a high level of trade credit to downstream channel partners (and the gains from franchising are enhanced), some of the transaction cost benefits of hierarchical governance, i.e., reliance on company-owned units (through buybacks), are likely to be relatively lower. Specifically, as we had argued earlier, one of the advantages of buybacks is that they allow franchisors to increase their bargaining power with franchisees to reduce transaction costs (Bradach, 1997; Michael, 2000). As firms are already investing in relationships with franchisees through higher provision of trade credit, these benefits of buybacks are likely to get tempered, which would reflect in lower shareholder gains from buybacks. Together, we posit:

##### H5a

 The positive effect of refranchising on stock returns of franchising firms is higher when trade credit provided by firms is higher.

##### H5b

 The positive effect of buybacks on stock returns of franchising firms is lower when trade credit provided by firms is higher.

It is well recognized by scholars that the performance impact of a firm’s strategic choices is influenced by environmental characteristics (Penrose, 1959). In keeping with extant marketing literature (e.g., Feng et al., 2017), we focus on munificence and dynamism dimensions in examining how industry characteristics moderate the effect of refranchising and buyback announcements on abnormal stock returns. In building our arguments, we rely on both agency theory and TCA as they underscore the role of environmental uncertainty as a source of governance problems between franchisors and franchisees.

#### Industry-level factor: Dynamism

Industry dynamism refers to the unpredictability of the sales environment in an industry (Dess & Beard, 1984). Agency theory arguments for franchising (e.g., Martin, 1988) suggest that the risk-sharing gains from refranchising existing company-owned units (where risk is entirely borne by the franchisor) should be greater when a franchise firm operates in a relatively more uncertain and dynamic environment. Additionally, Norton (1988) notes that the agency costs of monitoring company managers (with relatively underpowered incentives compared to franchisees) are higher in relatively dynamic environments (where it is easier to hide low effort). Further, when dynamism in the industry is high, the heightened uncertainty related to future market conditions can render managerial judgments and forecasts less reliable (Feng et al., 2017). In this regard, from a TCA perspective as well, Williamson (1981) expresses reservations about the relative benefits of hierarchical governance, noting potentially myopic control and dysfunctional outcomes in such settings. Based on this reasoning, the logic of the default TCA choice of markets (i.e., franchising) over hierarchies is enhanced in the presence of environmental dynamism. The TCA and agency theoretical support for franchising firms to use franchisees (rather than company-owned units) in more dynamic environments receives some empirical support in the franchising (e.g., Brickley & Dark, 1987) and broader marketing strategy (e.g., Klein, 1989) literatures as well. Therefore, we expect increased value relevance of refranchising (and decreased value relevance of buybacks) in industries characterized by higher levels of dynamism. Together, we posit:

##### H6a

 The positive effect of refranchising on stock returns of franchising firms is higher when the dynamism of the industry they operate in is higher.

##### **H6b**

The positive effect of buybacks on stock returns of franchising firms is lower when the dynamism of the industry they operate in is higher.

#### Industry-level factor: Munificence

Industry munificence reflects the capacity of an industry to support sustained organizational growth (Dess & Beard, 1984). When munificence is high, the overall sales in industry is growing fast, implying more growth avenues for firms. In such environments, agency theory suggests that the incremental benefits to the franchisors of risk sharing (Palmer & Wiseman, 1999) and of using the relatively stronger motivation (fueled by residual profit-sharing incentives) of an agent (franchisee) are diminished. Additionally, franchisors who rely heavily on company-owned and operated units can leverage the benefits of fiat to exploit emergent opportunities in munificent environments for their own cash flow gains, rather than sharing them with franchisees. Consistent with this reasoning, Geyskens et al. (2006) find that environmental munificence has a significant positive impact on hierarchical performance in their meta-analysis of TCA research. The above-mentioned agency theory and TCA theoretical arguments and empirical findings suggest a negative (positive) influence of refranchising (buybacks) on shareholder returns to firms in munificent industries. Together, we posit:

##### H7a

 The positive effect of refranchising on stock returns of franchising firms is lower when the munificence of the industry they operate in is higher.

##### H7b

 The positive effect of buybacks on stock returns of franchising firms is higher when the munificence of the industry they operate in is higher.

## Methodology

We use the event study methodology to examine the effects of franchising structure change (refranchising and buybacks) announcements on firm stock returns. This methodology relies on the efficient market hypothesis and offers a major advantage over other analytical techniques by allowing to directly test proposed cause-and-effect relationships between events of interest and stock price changes in a quasi-experimental setting (Sorescu et al., 2017; Srinivasan & Hanssens, 2009). The weak (and more accepted) form of efficient market hypothesis argues that “market, in which prices “fully reflect” all the public information, is efficient” (Fama, 1970, p.383). It is assumed that stock market prices incorporate all historical information about a firm, and no additional gains can be accrued by analyzing past stock market trends to predict future earnings. However, when novel and unexpected relevant information is generated, investors instantly update their expectations about future cash flows and adjust firm stock prices accordingly. The event study methodology holds validity by assuming that if, following an announcement, a security experiences a gain/loss beyond market expectations, the “abnormal” returns are attributable to the informational impact of the event of interest (Brown & Warner, 1985). Given our focus on refranchising and buyback announcements, it represents an appropriate methodology for our study. Specifically, by comparing the observed firm’s stock returns after buybacks or refranchising news is released with the expected stock returns, the event study methodology allows us to assess the shareholder impact of these strategic moves, while minimizing endogeneity concerns (Srinivasan & Hanssens, 2009).

### Data sources and sample selection

Our main unit of analysis is an announcement by a publicly traded firm owning franchising chain(s) to (a) refranchise previously company-owned and operated retail units or (b) buy back retail units from existing franchisees to operate them as company-owned units. To test our hypotheses, we use archival methods and bring together information from multiple data sources. First, we use the Bond’s Franchise Guide, Entrepreneur Magazine’s Franchise 500 Ranking, and FRANdata website to generate a list of 350 business-format franchising chains, which are either publicly-traded or owned by the publicly-traded firms. All three sources—Bond’s Franchise Guide (e.g., Jindal, 2011; Lafontaine & Shaw, 2005), Entrepreneur’s Franchise 500 Rankings (e.g., Lafontaine & Shaw, 2005; Shane et al., 2006), and FRANdata (e.g., Hsu et al., 2017)—are well-established sources extensively used in research and provide reliable annual information on U.S. franchisors. Next, we check for the completeness of franchising structure data as well as financials and ownership data for each firm in the sample because franchising chains are occasionally bought and sold by their parent firms. We ascertain the availability financial data needed for our main variables (as detailed below) in the COMPUSTAT database. If any of the required data was not available, we removed those firms/chains from the list. This step resulted in 102 franchising firms with the franchising structure and financial data in 2001–2020 needed for our analysis.

Second, to compile a sample of refranchising/buyback announcements for the franchising firms specified above, we perform a broad keyword search using FACTIVA and Nexis Uni, newswire services, annual reports, and corporate websites, for each year from 2001 to 2020. Corporate news often reaches markets via multiple channels, which warrants an examination of a variety of sources to ensure comprehensiveness in data collection and accuracy in detecting the dates of the first information release (Fotheringham & Wiles, 2022). The keywords utilized in our search were ‘refranchising,’ ‘buyback,’ ‘repurchase,’ ‘buyout,’ ‘chain growth,’ ‘conversion,’ ‘contract renewal,’ ‘contract termination,’ ‘ownership redirection,’ ‘vertical integration,’ ‘proportion of franchised outlets.’ Where there were ambiguities about the precise announcement date, we remove those announcements from the dataset. This step results in total 343 events (123 buybacks and 220 refranchising announcements).

Next, to minimize “noise” from potential confounding effects, we check for any contemporaneous announcements. Specifically, based on accepted practices in marketing research (e.g., Wiles et al., 2012), we control for financial (earning announcements, stock splits, stock buybacks) and strategy-related announcements (mergers and acquisitions, partnerships, joint ventures, lawsuits, executive management changes, and new product launches). When such announcements occurred within a two-trading-day window around the focal announcements, those contaminated events were removed from the sample. We also check if any of the collected announcements were a part of previously announced program to restructure firms’ franchising systems. During data collection, we collect details of why firms decided to pursue refranchising and buybacks and how they would be implemented (if such information is provided in the announcement). None of the collected announcements were identified as parts of previously announced restructuring programs. Further, our search provides confidence that none of the events were leaked to the public and investors before the event dates utilized in our study.

After accounting for confounding events, the final sample in our analysis includes 205 announcements (125 refranchising and 80 buybacks) made by 41 firms for 45 chains (see Figure WA.1 in the Web Appendix A). The sample is similar in size and composition to the samples used in other studies utilized publicly listed firms in the franchising context (i.e., Combs & Ketchen, 1999; Madanoglu et al., 2011; Hsu et al., 2017; Srinivasan, 2006). Web Appendix B (Tables WB 1 & WB 2) provides a yearly breakdown of firms announcing refranchising or buying back business units during the period of observations, along with the number of announcements made (refranchising vs. buybacks) by the firms every year.

### Measurement

#### Dependent variable

 The dependent variable in our study is the cumulative average short-term abnormal stock returns a firm accrues due to an announcement regarding a change in the structure of its franchising system (i.e., refranchise or buyback of downstream retail units). We gather information regarding the stock prices of the firms in the sample from the Center for Research in Security Prices (CRSP). We follow the recommendations by Sorescu et al. (2017) and apply the market-adjusted model (Brown & Warner, 1985) to calculate the cumulative average abnormal returns (*CAAR*_*i*_*)* of the firms in the sample. We also implement commonly accepted estimation process in marketing (e.g., Wiles et al., 2012) to calculate (*CAAR*_*i*_*)* for alternative event windows [*t*_*1*_*, t*_*2*_] within either side of the event date (see Web Appendix C for more details). To test the significance of the event windows and ensure that the results are not driven by influential events, we use a number of parametric and non-parametric tests, including parametric portfolio time-series deviation test, cross-sectional standard deviation test, and non-parametric generalized sign test in the analysis (Brown & Warner, 1985; Kothari & Warner, 2007). The statistical significance of these tests enables assessment of H1a&b. Subsequently, we use *CAAR* as the main dependent variable to evaluate hypotheses H2a&b through H7a&b.

#### Independent variables

We collect information from different sources to capture the predictor and control variables. Specifically, we rely on Bond’s Franchise Guide, Entrepreneur’s Franchise 500 Rankings, and FRANdata for variables related to franchising chains and COMPUSTAT for variables derived from annual accounting information disclosed by firms. All independent variables and controls are measured in the year prior to the announcement dates.

*Firm Royalty Rate (Royalty*_*i*_*):* Reflects the ongoing royalty paid by franchisees to the franchisor and is measured as a percentage of franchisee sales (Michael, 2002).

*Firm Advertising Intensity (AdvIntens *_*i*_*):* Reflects a firm’s advertising focus and operationalized as ratio of advertising expenses to firm sales (Combs & Ketchen, 2003).

*Firm Return on Assets* (*ROA*_*i*_*)*: Reflects firm’s asset utilization efficiency and operationalized as earnings before extraordinary items divided by total assets (Homburg et al., 2014).

*Firm Trade Credit Provided (TradeCredit*_*i*_*):* Reflects firm’s investments in downstream channel partners; operationalized as ratio of trade receivables to firm sales (Astvansh & Jindal, 2022).

*Industry Dynamism (IndDynamism*_*j*_*):* Reflects demand variation in an industry. We measure it as the standard error of the regression slope coefficient of the trend in industry sales divided by average sales in the industry over the past 5-years, with industry at the 4-digit SIC level (Dess & Beard, 1984).

*Industry Munificence (IndMunificence*_*j*_*):* Captures growth of demand in an industry. We operationalize it as the regression slope coefficient of the trend in industry sales by the average sales in the industry over the past 5-years, with industry at the 4-digit SIC level (Dess & Beard, 1984).

#### Control variables

  Additionally, we control for multiple firm and industry-level factors that may influence the stock market reaction to refranchising/buyback announcements, all measured in the year prior to the announcement dates. Specifically, at the firm level, we control for firm size and free cash flow. Larger firms are more likely to exploit economies of scale and scope to report better financial performance (Parsa et al., 2005). Further, free cash flow has been argued to affect shareholder value (Gruca & Rego, 2005). At the industry level, we control for the amount of competition in the industry by capturing industry concentration with the Herfindahl–Hirschman index. We also include the Food Retail sector dummy to control for fixed effects of the fast-food retail industry (Hsu et al., 2017) and yearly dummies to capture time fixed effects. Table 2 provides a summary of variables and data sources.

**Table 2:** Variables and data sources

Variable	Description	Source
Cumulative Abnormal Return CAAR_i_[t_1_, t_2_]	Firm's short-term abnormal stock returns	CRSP
Firm Royalty Rate (Royalty_i_)	Royalty paid by franchisees to the franchisor	Bond’s Franchise Guide, Entrepreneur Magazine’s Franchise 500 ranking, FRANdata website
Firm Advertising Intensity (AdvIntensity_i_)	Advertising expenses in relation to firm sales	COMPUSTAT
Firm Returns on Assets (ROA_i_)	Firm net income in relation to total assets	COMPUSTAT
Firm Trade Credit Provided (TradeCredit_i_)	Trade receivables in relations to firm sales	COMPUSTAT
Industry Dynamism (IndDynamism_j_)	Standard error of the regression slope coefficient in the sales trend divided by industry average sales of 5-year industry sales based on Dess and Beard (1984)	COMPUSTAT
Industry Munificence (IndMunificence_j_)	Regression slope coefficient in the sales trend divided by industry average sales of 5-year industry sales (Dess & Beard, 1984)	COMPUSTAT
	CONTROLS	
Firm Size (FirmSize_i_)	Firm total assets (ln)	COMPUSTAT
Firm Free Cash Flow (CashFlow_i_)	Operating cash flow in relation to total assets	COMPUSTAT
Industry Concentration (IndConcentration_j_)	Herfindahl–Hirschman index, a sum of squared market shares of all firms competing in the industry	COMPUSTAT
ADDITIONAL VARIABLES for the SELECTION MODEL		
Concept Development Time (ConcDevelopment_i_)	Number of years from chain inception to the year when it started franchising	Bond’s Franchise Guide, Entrepreneur Magazine’s Franchise 500 ranking, FRANdata website
Firm Financial Leverage (FinLeverage_i_)	Long-term debt to total assets	COMPUSTAT
Industry Sales (IndSales_j_)	Industry Sales (ln)	COMPUSTAT
Industry Growth (IndGrowth_j_)	Three-year average of industry sales growth rate (percentage)	COMPUSTAT

### Model specification

We estimate stock market reaction to refranchising or buyback announcements by calculating the cumulative average abnormal returns (CAAR) for our events of interest (Web Appendix C), Next, we implement cross-sectional analyses in two steps. First, it is possible that information not observable by investors drives the decision of a firm to refranchise or buyback (Kai & Prabhala, 2007). This may lead to selection bias in our sample as we only include those firms engaged in restructuring their franchising systems in the analysis. To safeguard against possible selection bias due to any potential systematic differences between the firms that franchise and undertake refranchising/buybacks decisions versus those that do not, we estimate the probability of a firm decision to refranchise/buyback retail units and calculate the inverse Mills ratio (IMR) (Web Appendix D). Next, we include the IMR as an additional firm-level control variable in the regression model shown below (Eq. 1) to evaluate our hypotheses for firm *i* in industry *j.* All the variables are as described earlier and in Table 2.

1 $$\begin{array}{c}{CAAR}_{i}[{t}_{1}, {t}_{2}]={\beta }_{0}+{\beta }_{1}{Royalty}_{i}+{\beta }_{2}{AdvIntensity}_{i}+{\beta }_{3}{ROA}_{i}+{\beta }_{4}{TradeCredit}_{i}+\\ {\beta }_{5}{IndDynamism}_{j}+{\beta }_{6}{IndMunificence}_{j}+{\beta }_{7}{FirmSize}_{i}+{\beta }_{8}{CashFlow}_{i}+\\ {\beta }_{9}{IndConcentration}_{j}+{\beta }_{10}{IMR}_{i}+{\beta }_{11}SIC5812Dummy+Yearcontrols+{\varepsilon }_{i}\end{array}$$

Although the event study methodology suffers from limited endogeneity concerns, we take some additional steps to ensure that the potential of endogeneity is further attenuated. Specifically, it is possible that shareholder returns to firm’s franchising structure decisions are endogenous with other firm characteristics, i.e., firm royalty rate, advertising intensity, ROA, and trade credit provided. Endogenous variables may be correlated with the error term, which violates the OLS assumptions, resulting in regression estimates that are unreliable (Wooldridge, 2002). To address the potential endogeneity issues, we apply the two-stage least squares (2SLS) methodology in estimating Eq. 1. The instrumental variables in 2SLS should be highly correlated with the endogenous variables (meet the relevance criteria) but have no direct effect on the dependent variable (the exclusion criteria). We follow the established practice in marketing (e.g., Germann et al., 2015) to instrument royalty rate, advertising intensity, ROA, and trade credit provided with the industry averages of these measures in the given, year excluding the focal firm, with industry defined at 4-digit SIC level. The proposed instruments are deemed appropriate as they meet the relevance criterion. This is because the focal firms face similar market conditions as their industry peers and it is reasonable to assume that their individual characteristics are correlated with the industry averages. Next, the instruments meet the exclusion criterion because the industry averages are unlikely to systematically impact individual firm’s financial performance and more specifically stock abnormal returns to refranchising/buyback announcements. We add industry average number of employees (minus the firm) and geographic dispersion (i.e., number of states in which the firm operates) as additional instruments, to meet the overidentification restriction criteria (Wooldridge, 2002). Hausman’s tests of endogeneity confirm that the 2SLS estimation approach is more appropriate and performs better than traditional OLS estimation. The postestimation analyses, specifically the first-stage regression results and the overidentification restrictions tests, confirm that the instruments are valid and the models perform consistently (Web Appendix E).

In addition, we take further steps to lower endogeneity concerns by following recommendations of Cameron and Miller (2015) for generating cluster-robust inferences for data sets with few clusters by clustering at the firm level. We also model year fixed effects. The decision to cluster at the firm level and include year fixed effects in the estimations is driven by following considerations. First, it can be argued that within any given year, clustering is due to shocks that are the same across all the observations in the year and can be effectively addressed by controlling for year fixed effects in the estimations. Second, clustering at the firm level produces a sufficient number of clusters, allowing for reliable cluster-robust inferences that account for cross-correlation and dependence across multiple observations for the same firm. We also control for the “eating places” industry through an indicator variable and include multiple industry factors, as main predictors (i.e., industry dynamism and munificence) and control (HHI). Finally, following previous research investigating two strategically opposite actions taken by firms (Wiles et al., 2012), we estimate Eq. (1) separately for refranchising and buybacks.

## Results

The dataset includes 205 announcements (125 refranchising and 80 buybacks) of 41 firms owning 45 franchising chains over the period from 2001 to 2020. The average number of announcements per firm is 5. The average firm in the dataset has a market capitalization of USD $1.3 billion. The dataset included firms in the industry sectors represented by 11 four-digit SIC codes, where 165 announcements (80%) fall under SIC 5812 (Food Retail Establishments), 24 announcements (11%) belong to SIC 7510 (Automotive Rentals and Leasing), and the rest (16 announcements) were approximately equally distributed across 9 industry sectors (see Web Appendix B for sample details). Table 3 provides a summary of the descriptive statistics.

**Table 3:** Descriptive statistics (main models)

	N = 205	1	2	3	4	5	6	7	8	9	10	11
1	CAAR (0; + 1)	1										
2	Firm Royalty Rate	-.021	1									
3	Firm Advertising Intensity	.088	-.033	1								
4	Firm ROA	-.135	.004	.154*	1							
5	Firm Trade Credit Provided	.024	.119	-.077	-.032	1						
6	Industry Dynamism	-.025	-.025	-.072	-.149*	-.032	1					
7	Industry Munificence	.115	-.144*	-.028	-.032	-.123	.504*	1				
8	Firm Size (log)	-.107	-.225*	-.038	.201*	-.062	.066	-.183*	1			
9	Free Cash Flow	-.002	-.083	.037	.529*	-.073	-.092	-.036	.021	1		
10	Industry Concentration	-.093	-.063	-.309*	-.185*	.134	.307*	.003	.151*	-.022	1	
11	Inverse Mills Ratio	-.010	.053	-.048	-.103	.281*	-.085	-.161*	.078	-.118	-.108	1
	Mean	.009	4.839	.03	.07	.77	.022	.047	7.181	.139	1282.049	3.345
	SD	.032	1.995	.021	.109	.011	.03	.064	1.581	.125	923.613	.739

*p < .05, (2-tailed tests of significance)

To assess the impact of the refranchising and buyback announcements on shareholder value, we estimate the short-term abnormal returns with the market-adjusted model and equally weighted index over alternative event windows 10 days around the day of announcement, using a combined dataset including both refranchising and buyback announcements (Table 4). The results show that on the day of the announcement, firms experience positive and statistically significant change in stock returns of 0.65% (*p* < *0.01*). Notably, the number of the events with a “positive” reaction significantly exceeds the number of the events with a “negative” reaction—118 vs. 87. This suggests that the effect is not driven by a few influential outliers but rather is due to the overall positive reaction of the stock market. Further, when calculating cumulative average abnormal returns (CAAR), we observe that on the one day after announcement, the positive cumulative effect reaches 0.92% (*p* < *0.01*) in the combined dataset (Table 5).

**Table 4:** Combined dataset: Daily abnormal returns for 10 days surrounding the event

Day	Observations	AAR	Positive: Negative	Portfolio Time Series CDA^a^	CSec Err t^a^	Generalized Sign Z^4^
-5	205	-.21%	100:105	-1.093	-.762	-.633
-4	205	.06%	107:98	.31	.231	.772
-3	205	-.22%	94:111	-1.129	-1.396$	-1.253
-2	205	-.04%	102:103	-.208	-.018	-.112
-1	205	-.08%	98:107	-.416	-.566	-.06
***0***	**205**	**.65%**	**118:87**	2.938***	2.639***	2.067*
1	205	.23%	112:93	1.184	1.732**	1.690**
2	205	-.09%	91:114	-.456	-.642	-1.048
3	205	.11%	107:98	.59	.164	.928
4	205	.07%	101:104	.365	1.032	.388
5	205	-.09%	104:101	-.456	-.914	-.053

^a^Portfolio Time Series CDA is a parametric test accounting for potential dependence of returns across security-events by estimating the standard deviation using the time series of sample (portfolio) mean returns from the estimation period (Warner and Brown 1985). CSec Err t is a standard parametric cross-sectional test that accounts for cross-sectionally correlated abnormal returns and heteroscedasticity in the abnormal returns. Generalized Sign Z is a nonparametric binomial test of whether the frequency of positive abnormal residuals is different from 0.5, which is well specified for event date variance increases and more powerful than the cross-sectional test (Cowan 1992)

^*^p < .1, **p < .05, ***p < *.01 (1-tailed tests of significance)*

**Table 5:** Combined dataset: Cumulative average abnormal stock returns (CAAR) over alternative event windows with market-adjusted model and equally weighted index

Day	Observations	CAAR	Positive: Negative	Portfolio Time Series CDA	CSec Err t	Generalized Sign Z
(-30,-2)	205	1.04%	97:108	.99	.627	-1.126
(-1, 0)	205	.65%	114:91	2.353***	2.369*	1.562
**(0, 0)**	**205**	**.65%**	**118:87**	3.337***	2.885**	2.166*
**(0, + 1)**	**205**	**.92%**	**124:81**	3.336***	3.656***	2.697**
(0, + 2)	205	.84%	112:93	2.505**	2.993**	1.531*
(0, + 3)	205	.99%	115:90	2.554**	2.991**	1.784*

^*^p < .1, **p < .05, ***p < *.01 (1-tailed tests of significance)*

Next, we examine the stock market abnormal returns separately for the refranchising and buyback announcements. In the refranchising subsample, on the day of the announcement, firms experience positive and statistically significant abnormal returns of 0.58% (*p* < *0.05*). On the following day, the cumulative abnormal returns to the refranchising announcements reach an average 0.75% (*p* < *0.01*), with 72 events reporting positive and 53 events revealing negative reactions in the stock market. In the buyback subsample, on the day of the announcement, firms experience positive abnormal returns equaling 0.75% (*p* < *0.05*) on average. On the one day after the announcements, the cumulative abnormal returns reach 1.18% (*p* < *0.01*), with “positive” events exceeding “negative” ones, 52 vs. 28 (Details are provided in Tables 6 and 7).

**Table 6:** Refranchising subsample: Cumulative average abnormal stock returns (CAAR) over alternative event windows with market-adjusted model and equally weighted index

Day	Observations	CAAR	Positive: Negative	Portfolio Time Series CDA	CSec Err t	Generalized Sign Z
(-30,-2)	125	2.02%	63:62	1.445*	1.112	.202
(-1, 0)	125	.67%	69:56	1.822**	2.042**	1.276
**(0, 0)**	**125**	**.58%**	**68:57**	2.231**	2.139**	**1.097**
**(0, + 1)**	**125**	**.75%**	**72:53**	2.035**	2.306**	1.812**
(0, + 2)	125	.65%	66:59	1.439*	1.676**	.739
(0, + 3)	125	.80%	67:58	1.546*	1.657**	.918

^*^p < .1, **p < .05, ***p < *.01 (1-tailed tests of significance)*

**Table 7:** Buyback subsample: Cumulative average abnormal stock returns (CAAR) over alternative event windows with market-adjusted model and equally weighted index

Day	Observations	CAAR	Positive: Negative	Portfolio Time Series CDA	CSec Err t	Generalized Sign Z
(-30,-2)	80	-.51%	34:46	-.334	-.49	-1.095
(-1, 0)	80	.61%	45:35	1.518*	1.255	1.365*
**(0, 0)**	**80**	**.75%**	**50:30**	2.658***	1.926**	2.484**
**(0, + 1)**	**80**	**1.18%**	**52:28**	2.937***	3.008***	2.931**
(0, + 2)	80	1.15%	46:34	2.334***	2.714***	1.589*
(0, + 3)	80	1.28%	48:32	2.267**	2.770***	2.036*

^*^p < .1, **p < .05, ***p < *.01 (1-tailed tests of significance)*

Across all the subsamples, both parametric and non-parametric tests are significant and consistent in sign, collectively lending support for H1a &1b and confirming that the results are robust to outliers. In addition, when we winsorize or remove the 1^st^ and 99^th^ percentile of the dataset and rerun the tests (Luo & Bhattacharya, 2009), we observe similar results.

Finally, we examine whether firms benefit more from refranchising or buyback strategies and implement mean difference tests for cumulative abnormal returns for the refranchising vs. buyback announcements over alternative event windows. For all the specifications and varying event windows, the results are nonsignificant, confirming that adjustments to the ownership structure of the franchising system (regardless of the direction of the change–refranchising vs. buyback) have a beneficial impact on firm stock returns, thus providing further support for H1a &1b (also see Figures WF.1 & 2 in Web Appendix F).

### Modeling contingency factors

We estimate the main models using the approach detailed above. To identify the most appropriate event window for cross-sectional analyses, we follow the common practice (e.g., Homburg et al., 2014; Geyskens et al., 2002; Sorescu et al., 2017) of using the event window that most completely captures the cumulative average abnormal returns (CAARs) (i.e., the event window that consistently reports the most significant t-test and z-test statistics) across various tests (i.e., parametric and non-parametric tests). The event window (0; + 1) demonstrated the most stable and consistently significant results across different model specifications, i.e., market-adjusted model vs. market model vs. Fama–French model vs. Fama–French-Carhart four-factor model, and across different periods of estimation. Therefore, we use CAARs over the event window (0; + 1), assessed with the market-adjusted model with the equally weighted index, as a dependent variable in the cross-sectional analyses. Our reliance on the market-adjusted model with equally weighted index to test the cross-sectional hypotheses is driven by prescriptions provided by marketing scholars for conducting event studies (Sorescu et al., 2017). To examine the specific drivers that shape the stock market response to the refranchising vs. buyback strategies, we estimate the model outlined in Eq. 1 separately for these events. We also check and observe that multicollinearity is not a major concern affecting our results. All the variance inflation factors are below 10 (Meyers et al., 2006), with average VIF_avg_ = 2.48 for the refranchising model and VIF_avg_ = 1.6 for the buybacks model.

### Refranchising model

Results show that the refranchising model, with the main predictors and control variables as outlined in Table 2, explains 40.7% of variance in CAARs in stock markets following the announcements and is significant at *p* < *0.001* level (see Table 8, Column (a)).

**Table 8:** Drivers of firm abnormal stock returns: Refranchising vs buyback announcements

Dependent variable: Abnormal Stock returns (0; + 1), Market-adjusted Benchmark	Hypotheses	Refranchising subsample (a)		Buyback subsample (b)	
		Coef	Robust Std. Err	Coef	Robust Std. Err
Firm Royalty Rate	H2a(-), b( +)	-.037*	.020	.017***	.007
Firm Advertising Intensity	H3a(-), b( +)	.100	.210	-.083	.377
Firm ROA	H4a(-), b( +)	-.170***	.080	-.054	.065
Firm Trade Credit Provided	H5a( +), b(-)	.067***	.030	.015	.026
Industry Dynamism	H6a( +), b(-)	.630	.470	.323	.289
Industry Munificence	H7a(-), b( +)	-.350***	.170	.240*	.132
Controls					
Firm Size (ln)		.005	.004	-.004	.004
Free Cash Flow		.096***	.047	.013	.038
Industry Concentration		.000	.000	.000	.000
Inverse Mills Ratio		-.002	.003	-.004	.010
SIC5812 dummy		.074***	.020	.035	.034
Year controls included in all specifications					
Intercept		-.032	.023	.010	.04
Observations		125		80	
Wald Chi2		1,950,000***		26,552.23***	
R square		.407		.341	

^*^p < .1, **p < .05, ***p < *.01 (2-tailed tests of significance)*

With respect to the individual predictors, we find that a firm’s royalty rate has a negative and marginally significant impact on firm’s abnormal stock returns caused by refranchising announcements, (*β* = -0.04; p-val < 0.1), providing partial support for H2a. However, we do not observe a significant effect of advertising intensity on stock market returns to refranchising announcements (β = 0.10; p-val < 0.50). As such, we do not observe support for H3a. In support of H4a, ROA has a significant negative effect (*β* = -0.17; p-val < 0.01) on stock returns following refranchising events. Finally, we observe that firm trade credit provided has a positive and significant effect (*β* = 0.07; p-val < 0.01) in shaping shareholder returns from refranchising decisions, supporting H5a.

At the industry level, industry munificence is seen to have a negative and significant impact (*β* = -0.35; p-val < 0.01), while industry dynamism has no effect on CAARs (*β* = 0.63; p-val < 0.17). As such, we find support for H7a, but not for H6a. Finally, with respect to controls, we find cash flows of firms to significantly increase the stock returns generated by refranchising. This may be because higher cash flows allow firms to attenuate some of negative agency and TCA related costs involved with franchising. We do not find firm size or industry concentration to have a significant effect, reflecting generalizability of our findings is not limited by the firm size or level of industry competition.

### Buybacks model

Next, we estimate the cross-sectional model for the buyback subsample (see Table 8, Column (b)). The results show that the model is significant at *p* < *0.001* level and explains 34.1% of variance in CAARs from the buyback announcements. However, the factors that drive abnormal returns in the buyback model differ strikingly from those in the refranchising model. More specifically, at the firm level, we only observe royalty rate to have a positive and significant effect (*β* = 0.02; p-val < 0.01) on abnormal stock returns from buyback announcements, confirming H2b. All other hypothesized firm-level factors are observed to have no significant effect, providing no support for H3b, H4b, and H5b. With respect to industry level factors, industry munificence has a positive and marginally significant effect on firm abnormal stock returns from buyback announcements (*β* = 0.24; p-val < 0.10), thus providing partial support for H7b. Yet, industry dynamism has no significant effect, rejecting H6b. Finally, with respect to controls, we do not find firm size and cash flows to impact firm stock returns generated from buybacks. This indicates that extra resources available to bigger firms and those with higher cash flows are not sufficient to attenuate some of the negative effects of buying back previously franchised units and bringing them within the boundaries of the firm. We also do not find industry concentration to have an effect, reflecting generalizability of our findings is not contingent on the level of competition in the industry.

### Additional robustness checks

To increase confidence in the results, we implement several robustness checks. We re-estimate the short-term abnormal returns with alternative benchmarks—the market model with equally weighted index (Brown & Warner, 1985) and Fama–French model (Fama & French, 1993), with alternative estimation periods of 300 days ending 30 days before the announcement day and 260 days ending 10 days before the announcement day (see Fama–French Model results in Web Appendix G). The CAARS on the day of the announcement and over (0; + 1) event windows remain positive and statistically significant across the subsamples and in the combined dataset (including refranchising/buyback announcements), providing added support for H1a&b.

Next, we conducted the Chow test for equality between the coefficients in the refranchising vs. buyback models. The Chow test allows us to examine whether the parameters for the refranchising subsample are equal to those for the buybacks subsample (Chow, 1960). The null hypothesis for the test is that there is no break point and the pooled data (refranchising and buybacks) can be represented with a single regression line. The results rejected the null hypothesis (F (7, 36) = 2.80, *p* < *0.01*), suggesting that the two groups (refranchising vs. buybacks) have different slopes and intercepts and cannot be pooled together. As such, the factors explaining stock market abnormal returns from refranchising announcements are different from those explaining from buyback announcements. We also re-estimate the cross-sectional models with the subsamples winsorized and trimmed at 1% levels and observe largely consistent results (see Web Appendix H). Next, as an additional robustness check, we drop the IMR variable and re-estimate the main models. All the hypothesized relationships hold in terms of the direction and significance (Web Appendix I). Together, these checks increase confidence in our overall findings.

Finally, we check if firms experience any long-term returns to refranchising/buyback announcements, using the long-horizon event study methodology (Kothari & Warner, 2007). For long-term returns, extant literature suggests two alternative approaches, the buy-and-hold abnormal returns approach and the calendar-time portfolio returns with the Fama–French benchmark and a 1-year horizon (Kolari & Pynnönen, 2010). Each of the methods has strengths and limitations (Kothari & Warner, 2007; Srinivasan & Hanssens, 2009). To ensure the robustness of the results, we utilize both approaches. None of the specifications yield statistically significant results, suggesting that firms do not accrue any long-term returns from refranchising and buybacks. These results support the argument that corporate news like refranchising and buybacks provide strong economic information to financial markets about firm strategies. Since such events are costly and less reversible, they provide credible signals about a firm’s commitment towards a strategic direction, effectively reducing the level of uncertainty investors might have about the firm and enabling accurate assessment of its future growth prospects. This assessment gets incorporated into firm stock market price in the short-term and does not require a long-term assessment in the financial markets. It, therefore, appears that in the context of refranchising/buyback announcements, stock markets remain efficient, rendering the short-term event study methodology appropriate for our analysis.

## Discussion

Distribution channels are important elements of a firm’s marketing mix. In this study, we draw insights from the efficient contracting perspective, which encompasses agency theory and transaction cost analysis (TCA), to focus on the shareholder value implications of franchising channel structure decisions made by firms. We utilize the event study methodology to show that announcements of both refranchising and buybacks of downstream retail units by franchising firms increase their stock returns. Furthermore, we evaluate the role of theoretically derived firm- and industry-level factors in moderating the shareholder returns from refranchising and buybacks announcements by the firms. Together, the findings (and the theoretical framework) contribute to scholarly research in marketing. Further, they offer some actionable guidance for managers regarding their franchising strategies.

### Theoretical contributions

Our study makes multiple contributions to marketing theory. Although extant research offers rich insights into channel structures (e.g., Lafontaine & Kaufmann, 1994), channel governance (e.g., Bergen et al., 1992), channel additions (Geyskens et al., 2002; Homburg et al., 2014), channel deletions (e.g., Kumar, 2021), and channel management (e.g., Palmatier et al., 2020), evidence for financial implications of distribution channel related strategic decisions is relatively limited (e.g., Gielens & Geyskens, 2012). Particular to franchising, some studies have compared the differences in financial performance between franchising and non-franchising firms (e.g., Madanoglu et al., 2011), and others have considered the financial effects of dual distribution structures, i.e., having a mix of franchise-owned and company-owned retail units (e.g., Srinivasan, 2006). Yet, the impact of changes in levels of franchising (and the direction of these changes), while keeping the size of the distribution chain constant, on shareholder value of firms remains to be examined in detail. Indeed, to the best of our knowledge, only one study has considered the impact of changes in franchising proportion on stock market returns (Hsu et al., 2017), and we complement and go beyond their findings in substantive and meaningful ways. We discuss our contributions in more detail next.

First, our reliance on the efficient contracting perspective (i.e., agency theory and transaction cost analysis (TCA)), along with the use of the event study methodology enabled us to articulate and evaluate the causal effects of changes in franchising structure (in terms of refranchising and buybacks) on shareholder value of franchising firms.^3^ In particular, we theoretically illustrate the downsides and benefits of refranchising and buybacks, two seemingly opposite strategies, for franchising firms. Our analysis confirms that financial markets reward firms for both refranchising and buybacks decisions. There has been a considerable debate regarding the optimal proportion of franchise ownership for firms, with some studies asserting that firms are likely to favor franchising initially but then move towards company ownership of retail units, whereas others arguing the opposite (Dant & Kaufmann, 2003). In squarely responding to this debate, we underscore that, from a shareholder value perspective, neither of the two opposing arguments stand rejected. Instead, financial markets align with the “Darwinian economics” rationale (Anderson, 1988), supporting the need for firms to react to competitive market forces in a manner that best leverages their situation. Thus, firms may select opposing strategies (in terms of hierarchical or market-based governance) to optimize their performance.

Second, by investigating the impact of opposing strategies in the context of franchising, we add to the limited work in marketing strategy validating the shareholder value of apparently conflicting strategic moves by firms based on contingent factors (e.g., Wiles et al., 2012). It also allows us to go beyond Hsu et al. (2017), who examined annual changes rather than discrete and specific events involving changes in franchise ownership structure and did not offer visibility into the separate effects of refranchising and buybacks on firm stock returns. Furthermore, our framework and empirical methodology provides a template for understanding other firm decisions where agency theory and TCA prescriptions are at play in the determination of the organization of economic activity within or outside the boundaries of the firm. For instance, decisions involving in-house sourcing vs. outsourcing (for a range of business functions such as production, advertising, marketing research, salesforce etc.), licensing vs. owning facilities in international markets, and offshoring vs. in-shoring are few examples of opposing strategies that have both benefits and downsides for firms. Our approach of utilizing an efficient contracting perspective (e.g., Bergen et al., 1992; Rindfleisch & Heide, 1997), in combination with the event study methodology, can be extended to evaluate the shareholder value implications of these strategic choices made by firms.

Third, we build on the assumptions of agency theory and TCA to identify and articulate boundary conditions imposed by firm and industry factors on the shareholder value impact of franchising decisions (refranchising and buybacks). Both theories alert to the potential of moral hazard and opportunism in the franchisee-franchisor relationship and the costs involved in mitigating these exchange hazards. They also highlight the impact of environmental uncertainty on the hierarchical and vertical governance structures utilized by firms. We draw from these views to present a nuanced picture of the forces shaping the boundary conditions in our study.

Specifically, based on agency theory we find that a firm’s royalty rate has an attenuating/enhancing effect on the incremental shareholder value derived by firms from refranchising/buybacks. This supports the argument that as firms with higher royalty rates face greater risks of free-riding and moral hazard by franchisees, the gains accruing to them from refranchising get attenuated and firms are better placed to buy back some of their existing franchised units. Along similar lines, we had built on agency theory to argue franchising firms with high advertising intensity would also derive lower/higher stock returns from refranchising/buybacks. Our predictions were driven by observations that higher advertising intensity reflects advertising as a central element of a firm’s marketing strategy and the firm’s investment in building customer-based resources. In such, cases, heightened agency issues associated with franchisees would reduce the gains accruing to the firm. However, our results did not provide confirmation for these hypotheses. It is possible that the local market knowledge of franchisees allows firms to better target their advertising efforts (an aspect not observable in our data), generating consumer surplus which may be overcoming the agency costs associated with working with them. Similarly, we had posited that firms would be able to appropriate the higher value generated by advertising for themselves in the case of buybacks, instead of having to share this with franchisees. As such, firms with higher advertising intensity were predicted to benefit more from buybacks. However, results did not confirm this prediction as well, reflecting that the consumer surplus generated from advertising, without the benefits of local know-how afforded by franchisees, may not be sufficient to cover the acquisition costs and other agency issues associated with hierarchical governance.

A similarly complex set of findings emerge for the two firm-level moderating factors motivated by a TCA lens—ROA and trade credit provided. Consistent with the prediction, results support the argument that firms with high ROA would benefit less from refranchising, given that high ROA reflects lower need for firms to rely on outside partners to alleviate their resource scarcity. Further, as predicted firms that provide higher trade credit to their channel partners, reflecting investments in stronger channel partnerships, appear to derive greater shareholder benefits from refranchising. For buybacks, we do not find support for these factors, possibly due to positive and negative forces balancing each other out.

Finally, our study draws attention towards the boundary conditions created by the industry environment faced by firms. We find that industry munificence weakens the beneficial effect of refranchising on shareholder value, while marginally elevating the stock returns derived from buybacks. This supports our position that when the industry environment offers more growth opportunities, it may be possible for firms to do well operationally without reliance on partners (franchisees). Further, with buybacks, firms will be able to capture more of the overall industry growth for their own cash flow gains, enhancing the shareholder value effects. In dynamic industries, agency theory and TCA led us to argue that greater reliance on franchisors would be beneficial for firms. However, our analysis didn’t confirm these hypotheses, indicating industry dynamism as not a value driver for firms making refranchising and buybacks moves.

### Managerial contributions

There have been numerous calls for researchers to show how marketing strategy decisions contribute to shareholder value (e.g., Srivastava et al., 1998). In adding to the marketing-finance interface literature that has emerged in response to these calls, we assess the impact of franchising decisions on firm stock returns. As we outlined previously, franchising is an important form of distribution strategy, which contributes substantially to the economy both in terms of economic output and employment. Furthermore, by focusing on franchising as a specific context, we are able to provide more customized insights to managers in franchising firms, compared to what generalized studies would be able to offer (Stremersch et al., 2022). Specifically, our research answers several key questions faced by managers of franchising firms considering structural changes to their distribution channels, while keeping the size of their distribution chain size constant. Moreover, we highlight that different boundary conditions govern the firm stock returns derived from refranchising and buybacks, providing nuanced guidance to franchisors.

#### Do managers benefit shareholders from changes to franchising structures?

One of the fundamental lessons from the marketing literature is that managers should stay alert to changing market conditions and regularly recalibrate their marketing mix. We provide empirical support for this prescription by showing that, when it comes to franchising, managers benefit shareholders by changing their franchising levels at discrete intervals. Indeed, within the confines of our sample, we observe that investors in the U.S. reward shareholders of franchising firms announcing refranchising and buybacks of retail units by a median amount of $8.0 million and $8.1 million respectively on the day of announcement, with the median gains rising to as much as $10.3 million and $12.8 million respectively one day after the announcement.

#### When can managers derive greater benefits for their shareholders from refranchising?

In addition, our study reveals that the stock market attaches higher value to firms deciding to refranchise if they are in a position to charge lower royalty rates and ROA and provide higher trade credit to their downstream partners. Our results reveal that managers who set low royalty rates generate $79.6 million additional median gains to their shareholder from refranchising, as they stand to lose less from potential moral hazard. Further, firms that have low ROA also benefit shareholders more from refranchising decisions (to the tune of an additional median $7.0 million), deriving benefits from the local market knowledge and access brought to them by the franchisees. Finally, firms that invest more in building relationships with downstream partners by providing them with higher trade credit also generate $71.0 million in median gains from refranchising. Together, these present actionable insights to managers to benefits their firms from refranchising their existing retail units.

#### When can managers benefit their shareholders more from buybacks?

We further underscore that financial gains from buybacks are elevated if firms have higher royalty rate. In particular, based on our sample and empirical estimates, firms with higher royalty rates stand to gain $36.6 million from buybacks, leading to overall median gains of $44.7 million to shareholders. Managers should take note of this and undertake more buybacks in situations where their brands and other customer-based assets are more vulnerable to misappropriation from franchises.

#### Should managers take industry conditions into account when formulating DVI strategy?

With respect to industry conditions, we show that managers in rapidly growing industries stand to gain less and more from refranchising and buybacks respectively, than those in stable industries. It appears that in munificent industries, it may be possible for managers to generate $12.4 million more in gains to shareholders from buybacks, bringing the total median gains to $20.5 million. In contrast, shareholders stand to gain $22.2 million if their firms refranchise in lower growth industries, bringing overall gains of $30.2 million to shareholders.

### Limitations and directions for future research

Our research also suffers from some key limitations that suggest useful areas for further inquiry. In deriving our framework, we outlined key boundary conditions but our inquiry was limited to factors observable through secondary/archival data. As such, we were able to only indirectly capture certain theoretically relevant moderators like management supervision, quality control, and brand reputation through ROA, trade credit provided, and royalty rate respectively. Future studies can directly measure these constructs through primary research, such as managerial surveys. In addition, product-demand and channel-demand growth (as studied by Geyskens et al., 2002) as well as order of entry of firms in the industry may influence investor reactions to refranchising announcements. However, we do not have product and channel-level demand data for our sample. Future work can investigate the role of these boundary conditions, if the required data becomes available. Additionally, our sample was restricted to relatively larger, public-traded firms. However, the franchising industry has a large number of smaller and privately held firms, not as well represented in some of the secondary data sources. We observe firm size to be a weak boundary condition in our analysis, indicating that our results are likely to be generalizable to smaller firms. Further research can apply our framework through primary data collection techniques to provide additional confidence in the usefulness of our findings for managers across a larger spectrum of franchising firms. Finally, we restricted our context to franchising, which offers certain benefits in terms of pointed and directly useful findings for franchising managers (Stremersch et al., 2022). We submit that our theoretically derived framework and event study methodology should be applicable to other contexts as well and scholars can build on our study to evaluate these contexts in future research to enhance the external generalizability of our findings.

### Supplementary Information

Below is the link to the electronic supplementary material.

Supplementary file1 (DOCX 91.5 KB)

## Footnotes

1 We define refranchising as a change in franchising structure, where a firm increases its share of franchised stores by selling its company owned units to franchisees, while keeping its distribution coverage constant. In contrast, a buyback represents a change in franchising structure, where a firm buys back previously franchised units to increase its share of company owned outlets, while its keeping distribution coverage constant.

2 We recognize there are different types of franchising formats. We focus on business format franchising, where firms license their brand names and ways of doing business to franchisees in return of royalties (Lafontaine, 1992).

3 We thank the review team for guiding us to evaluate refranchising and buyback decisions separately.
