# Supplementary material for: Franchising structure changes and shareholder value: Evidence from store buybacks and refranchising
Source: J Acad Mark Sci. 2023 Jan 14;51(5):1098–117. doi: 10.1007/s11747-022-00921-3 (PMC10435657; doi:10.1007/s11747-022-00921-3)
Supplement: Supplementary file 1 — Supplementary file1 (DOCX 91.5 KB) [file 11747_2022_921_MOESM1_ESM.docx]

**Web appendix A.**

**WA.1: Data sources and collection process**

Bond’s Franchise Guide

Entrepreneur Ranking 500

FRANdata (2001-2020)

Factiva

NexisUni Academic

Newswire Services

Company websites

Keyword search

Sample of 205 announcements (125 refranchising and 80 buybacks)

102 publicly-traded franchisors

343 announcements: Exact dates of first information release. Removal of contaminated events

CRSP

COMPUSTAT

Firm Annual Reports

**Examples of Refranchising and Buyback Announcements**

|  | Firm | Chain | Event |
| --- | --- | --- | --- |
| Refranchising | CKE Restaurants | Hardee's | *CKE Restaurants, parent company of Hardee's Food Systems said it sold 30 Hardee's restaurants in the Kansas City area (Dec 5, 2007)* |
|  | Wendy's | Wendy's | *Wendy's Sells 30 More Locations...Bridgeman, one of the restaurant industry’s most established athlete franchisees, bought 30 Wendy’s units in the St. Louis market through BB St. Louis Inc. (Aug 13, 2013)* |
| Buybacks | Brinker | Chili's | *Brinker International is buying 116 Chili's restaurants from a franchise operator. Investors should expect the deal to improve earnings (July 10, 2019)* |
|  | Sonic | Sonic | *Sonic to buy 73 units from bankrupt franchisee (Feb 3, 2017)* |

**Web appendix B**

**Sample details**

**Table WB 1. Firms refranchising/ buying back units in 2001-2020**

| Year | Refranchising Firms | Buying Back Firms |
| --- | --- | --- |
| 2001 | New Horizons Worldwide Inc; Famous Dave’s of America Inc; Friendly Ice Cream Corp | Checkers Drive in Rstrnts Inc; Krispy Kreme Doughnuts Inc; Sonic Corp |
| 2002 | AFC Enterprises Inc | Applebee’s International Inc; Krispy Kreme Doughnuts Inc; Triarc Companies Inc |
| 2003 | Marriott International Inc; Triarc Companies Inc | Applebee’s International Inc; Dollar Thrifty Auto Grp; Krispy Kreme Doughnuts Inc; Outback Steakhouse Inc; Panera Bread Co; Roto Rooter Inc |
| 2004 | Friendly Ice Cream Corp; 7 Eleven Inc; CKE Restaurants Inc; IHOP Corp | ACE Cash Express Inc; Applebee’s International Inc; Dollar Thrifty Auto Grp; Sonic Corp |
| 2005 | Hilton Hotels Corp | Aaron Rents Inc; Applebee’s International Inc; Dollar Thrifty Auto Grp; Option Care Inc; Panera Bread Co; Sonic Corp; Triarc Companies Inc |
| 2006 | Jack in The Box Inc; CKE Restaurants Inc; Yum Brands Inc | AFC Enterprises Inc; Benihana Inc; Checkers Drive in Rstrnts Inc; Dollar Thrifty Automotive Grp; Red Robin Gourmet Burgers Inc; Rubio’s; Westaff Inc |
| 2007 | Hilton Hotels Corp; Brinker International Inc; Buffalo Wild Wings Inc; CKE Restaurants Inc; Brooke Corp | Dollar Thrifty Automotive Grp; Panera Bread Co; Red Robin Gourmet Burgers Inc |
| 2008 | CKE Restaurants Inc; IHOP Corp; Jack in The Box Inc | Aaron Rents Inc; Burger King; Triarc Companies Inc |
| 2009 | Brinker International Inc; Burger King; Dollar Thrifty Auto Grp; Yum Brands Inc | Wendy’s Co |
| 2010 | Burger King; Jack in The Box Inc; Dineequity |  |
| 2011 | Berkshire Hathaway Inc; Buffalo Wild Wings Inc; Domino’s Pizza Inc; Dineequity Inc; Yum Brands Inc |  |
| 2012 | AFC Enterprises Inc; Buffalo Wild Wings Inc; Dineequity Inc; Jack in The Box Inc; Yum Brands Inc | Red Robin Gourmet Burgers Inc |
| 2013 | Burger King; Jack in The Box Inc; Dineequity Inc; Krispy Kreme Doughnuts Inc; Wendy’s Co |  |
| 2014 | Jamba Inc; Krispy Kreme Doughnuts Inc; Wendy’s Co | Burger King; Red Robin Gourmet Burgers Inc |
| 2015 | Buffalo Wild Wings Inc; Jamba Inc; Krispy Kreme Doughnuts Inc; McDonalds Corp; Panera Bread Co; Wendy's Co; Yum Brands | Buffalo Wild Wings Inc; McDonalds Corp |
| 2016 | Bloomin Brands Inc; Burger King; Dineequity Inc; Jamba Inc; Krispy Kreme Doughnuts Inc; Panera Bread Co; Papa Murphy’s Holdings Inc; Yum Brands Inc Wendy's Co |  |
| 2017 | Aaron Rents Inc; Bloomin Brands Inc; Buffalo Wild Wings Inc; Jamba Inc; McDonalds Corp; Sonic Corp; Yum Brands Inc | Aaron Rents Inc; McDonalds Corp; Sonic Corp |
| 2018 | Papa Murphy’s Holdings Inc; Yum Brands Inc | Dineequity Inc |
| 2019 | Dineequity Inc; Red Robin Gourmet Burgers Inc; Yum Brands Inc | Brinker International Inc; Burger King; Chemed Corp New; Domino’s Pizza Inc |
| 2020 |  | McDonalds Corp; Wendy’s Co |

**Table WB 2. Refranchising/buyback announcements by year: 2001-2020**

| Year | Refranchising Announcements | Buyback  Announcements | Totals |
| --- | --- | --- | --- |
| 2001 | 4 | 5 | 9 |
| 2002 | 1 | 4 | 5 |
| 2003 | 3 | 11 | 14 |
| 2004 | 4 | 6 | 10 |
| 2005 | 1 | 9 | 10 |
| 2006 | 3 | 12 | 15 |
| 2007 | 7 | 10 | 17 |
| 2008 | 4 | 3 | 7 |
| 2009 | 5 | 1 | 6 |
| 2010 | 10 | 0 | 10 |
| 2011 | 6 | 2 | 8 |
| 2012 | 9 | 1 | 10 |
| 2013 | 12 | 0 | 12 |
| 2014 | 4 | 3 | 7 |
| 2015 | 13 | 2 | 15 |
| 2016 | 13 | 0 | 13 |
| 2017 | 11 | 3 | 14 |
| 2018 | 7 | 1 | 8 |
| 2019 | 8 | 5 | 13 |
| 2020 | 0 | 2 | 2 |
| **Total** | **125** | **80** | **205** |

**Table WB 3. Industry sectors in the study**

| SIC code | Description | N |
| --- | --- | --- |
| 5412 | Food Stores | 2 |
| 5812 | Eating Places | 165 |
| 6099 | Depository Banking | 1 |
| 6331 | Fire, Marine, and Casualty Insurance | 2 |
| 6794 | Miscellaneous Investment Offices | 2 |
| 7011 | Hotels and Motels | 1 |
| 7359 | Equipment Rentals and Leasing | 3 |
| 7363 | Help Supply Services | 2 |
| 7510 | Automotive Repair, Services, and Parking | 24 |
| 7600 | Miscellaneous Repair Services | 1 |
| 8082 | Healthcare Services | 2 |

**Web Appendix C**

**Calculation of cumulative average abnormal returns**

First we estimate the expected returns *E(R_it_* *)* of firm *i* on day *t* based on the history of stock returns and general market trends (eq. 1):

*E(R_it_* *)=R_mt_  (1),*

Where, *R_mt_ – is the average rate of return of all stocks trading in the stock market at time t.*

Following established practice, we assume that no information regarding the event of interest was released during the estimation period - this was confirmed through our search of the different news sources available to investors as described earlier.

Second, we compute short-term abnormal returns (*AR_it_*) (see eq. 2) as the difference between actual returns *R_it_* and expected returns *E(R_it_* *)* on the event day:

*AR_it_= R_it_ - E(R_it_* *) (2)*

Third, we calculate the cumulative abnormal returns *CAR_i_* [*t_1_, t_2_*] by aggregating daily abnormal returns over event windows [*t_1_, t_2_*] within 10 days of both sides of the announcement day to control for information leakage and delayed stock market reaction to announcements:

*CAR_i_* [*t_1_, t_2_*] =$\sum_{t1}^{t2}$AR*_it_  (3)*

Finally, in Equation 4 we calculate the cumulative average abnormal returns (*CAAR_i_)* of the firms in the sample for alternative event windows [*t_1_, t_2_*] within either side of the event day:

*CAAR_i_*=Σ (*CAR_i_* [*t_1_, t_2_*]/n) *(4)*

**Web appendix D: Heckman selection model**

Firms make a deliberate strategic decision to engage in refranchising or buyback of retail units based on private information that is seldom fully known to investors. To capture the effects of unobservable private information, we follow the recommendations of Sorescu et al. (2017) and apply the Heckman procedure (Heckman 1979) to estimate a firm’s decision to refranchise or buyback as a function of firm and industry-specific factors.

An important consideration when designing the Heckman selection procedure is whether to add exclusion restrictions to the model - factors that drive the decision to select a strategy but are unlikely to impact outcomes. In principle, some scholars indicate that an exclusion criterion is not strictly necessary in the Heckman model because it is identified by non-linearity (Kai and Prabhala, 2007; Sorescu et al. 2017). However, conservatively and to eliminate possible near-multicollinearity issues (Kai and Prabhala 2007), we include Concept Development Time (*ConcDevelopment_i_)* as one of the exclusion criteria. Concept development time relates to the number of years that a franchisor has been in business, developing the business concept, before it decides to franchise. It is like that the longer a firm is in business before its starts to franchise, the more attuned it becomes to the benefits and shortcomings of the franchising model for its business format (Shane 1996). This can affect firm’s confidence in making franchising structure change decisions and make it more likely to make such decisions. This makes the *Concept Development Time* a suitable exclusion criterion for our model (Hamilton and Nickerson 2003). In addition, we control for firm financial leverage *(FinLeverage_i_)* because firm’s access to capital is likely to affect its future strategy choices (Malshe and Agarwal 2013; Oxenfeldt and Kelly 1968). Specifically, in the context of franchising, higher leverage can make firms rely more on franchising, leading them to refranchise existing retail stores. In contrast, higher leverage can restrict firm’s ability to acquire the capital to buyback some of its franchised stores.

At the industry level, we include industry size *(IndSales_j_)* and three-year average industry growth (*IndGrowth_j_*) to control for overall industry demand. Companies operating in smaller and lower growth industries might find it difficult to acquire support from external investors for significant structure changes and would have limited opportunities to make such changes. As such, they will be less likely engage in franchising structure changes. These characteristics make these industry level variables satisfy exclusion criterion for the selection model (Hamilton and Nickerson 2003). Finally, to account for the environmental and time fixed effects, we include industry fixed effects at 4-digit SIC code level and year fixed effects.

*Decision to Refranchise/Buy Back in year (t+1) = β_0_+ β_1_ConcDevelopment_i_+ β_2_FinLeverage_i_ +β_3_IndSales_j_ + β_4_IndGrowth_j_ + industry & year controls +t + ε_it_*

**Table WD.1: Selection model results**

| Decision to refranchise/buy back units | Coef. | Robust St. Err |
| --- | --- | --- |
| Concept Development Time | .070*** | .011 |
| Firm Financial Leverage | -.019 | .069 |
| Industry Sales (ln) | -.187* | .101 |
| Industry Growth (3Y-average) | .155 | 1.000 |
| Industry and Year controls are included | |  |
| Intercept | -3.277** | 1.172 |
| Wald Chi2 | 104.49*** |  |
| N | 5780 |  |

**p<.1, **p<.05, ***p<.01, (2-tailed tests of significance)*

**Table WD.2: Descriptive statistics (selection model)**

|  | N=5780 | 1 | 2 | 3 | 4 | 5 |
| --- | --- | --- | --- | --- | --- | --- |
| 1 | Decision to refranchise/buy back | 1 |  |  |  |  |
| 2 | Firm Concept Development Time | .162* | 1 |  |  |  |
| 3 | Firm Financial Leverage | .001 | .002 | 1 |  |  |
| 5 | Industry sales (ln) | .012 | .012 | -.050* | 1 |  |
| 6 | Industry Growth (3Y-average) | -.002 | .008 | -.007 | .061* | 1 |
|  | Mean | .019 | 1.519 | .376 | 1.985 | .042 |
|  | SD | .137 | 6.156 | 5.046 | 1.712 | .08 |

**p<.05, (2-tailed tests of significance)*

Following previous research (e.g., Wiles et al. 2012), we calculate the inverse Mills ratio (*IMR_i_*) based on this selection model and use it as a control in the main model.

As an additional precautionary measure, we estimate the variance inflation factors (VIF) to check for the multicollinearity issues in the selection model (Kai and Prabhala 2007). All the VIFs are well below 10 (Meyers et al. 2006), with average VIF_avg_ = 1.38. Thus, multicollinearity is not a major concern.

Finally, the selection model has a larger sample size (compared to the sample utilized in the main analysis) due to multiple reasons. First, the selection model includes all retailers and not all of them franchise. Second, even among retailers that franchise, many don’t make franchising structure changes every year. Finally, our main sample excluded buybacks/refranchising with potentially confounding events, further reducing the sample size.

**Web Appendix E**

**Table E1. Refranchising subsample. First-stage summary regression statistics**

|  |  | Adjusted | Partial | Robust |  |
| --- | --- | --- | --- | --- | --- |
| Variable | R sq | R-sq. | R-sq. | F (6,28) | Prob>F |
| Firm Royalty Rate | .990 | .986 | .976 | 186.362 | .000 |
| Firm Advertising Intensity | .743 | .657 | .406 | 2.493 | .047 |
| Firm ROA | .648 | .530 | .150 | 9.837 | .000 |
| Firm Trade Credit Provided | .846 | .794 | .727 | 9.469 | .000 |

**p<.1, **p<.05, ***p<.01 (2-tailed tests of significance)*

**Tests of overidentifying restrictions**:

| Sargan chi2(2) = 1.969 | (*p = .374*) |
| --- | --- |
| Basmann chi2(2) = 1.489 | (*p = .475*) |

**Table E2. Buybacks subsample. First-stage summary regression statistics**

|  |  | Adjusted | Partial | Robust |  |
| --- | --- | --- | --- | --- | --- |
| Variable | R sq | R-sq. | R-sq. | F(6,23) | Prob>F |
| Firm Royalty Rate | .857 | .775 | .714 | 15.522 | .000 |
| Firm Advertising Intensity | .845 | .755 | .579 | 8.658 | .000 |
| Firm ROA | .791 | .670 | .420 | 4.823 | .003 |
| Firm Trade Credit Provided | .992 | .987 | .989 | 161.870 | .000 |

**p<.1, **p<.05, ***p<.01 (2-tailed tests of significance)*

**Tests of overidentifying restrictions**:

| Sargan chi2(2) = 2.914 | *(p=0.233)* |
| --- | --- |
| Basmann chi2(2) = 1.890 | *(p=0.389)* |

**Web appendix F: Figures for returns 10 Days surrounding the announcements**

**Figure WF.1: Refranchising subsample: Daily average abnormal returns (AAR) and cumulative abnormal returns (CAAR) for 10 days surrounding the announcement to refranchise business units.**

**Figure WF.2: Buybacks subsample: Daily average abnormal returns (AAR) and cumulative abnormal returns (CAAR) for 10 days surrounding the announcement to buy business units back from franchisees.**

**Web Appendix G**

**Table G.1 CAAR estimated with Fama-French benchmark with equally weighted index over 300 days ending 30 days before the event (combined dataset)**

| Day | Observations | CAAR | Positive: Negative | Portfolio Time Series CDA | CSec Err t | Generalized Sign Z |
| --- | --- | --- | --- | --- | --- | --- |
|  |  |  |  |  |  |  |
|  |  |  |  |  |  |  |
| (-30,-2) | 205 | -.16% | 91:114 | -.14 | -.144 | -1.368* |
| (-1,0) | 205 | .51% | 114:91 | 1.716** | 2.060** | 1.808** |
| **(0, 0)** | **205** | **.59%** | **120:85** | **2.805***** | **3.003***** | **2.637***** |
| **(0,+1)** | **205** | **.64%** | **122:83** | **2.135***** | **2.759***** | **2.913***** |
| (0,+2) | 205 | .56% | 117:88 | 1.525* | 2.136** | 2.223** |
| (0,+3) | 205 | .71% | 120:85 | 1.681** | 2.472*** | 2.637** |

**p<.1, **p<.05, ***p<.01 (1-tailed tests of significance)*

**Table G.2 CAAR estimated with Fama-French benchmark with equally weighted index over 300 days ending 30 days before the event (refranchised subsample)**

| Day | Observations | CAAR | Positive: Negative | Portfolio Time Series CDA | CSec Err t | Generalized Sign Z |
| --- | --- | --- | --- | --- | --- | --- |
|  |  |  |  |  |  |  |
|  |  |  |  |  |  |  |
| (-30,-2) | 125 | 1.42% | 61:64 | .902 | .842 | -.029 |
| (-1,0) | 125 | .46% | 70:52 | 1.126 | 1.591* | 1.570* |
| **(0, 0)** | **125** | **.48%** | **68:57** | **1.645*** | **2.077**** | **1.214*** |
| **(0,+1)** | **125** | **.49%** | **70:55** | **1.191*** | **1.821**** | **1.570*** |
| (0,+2) | 125 | .44% | 68:57 | .877 | 1.454* | 1.214 |
| (0,+3) | 125 | .64% | 73:52 | 1.102 | 1.846** | 2.102** |

**p<.1, **p<.05, ***p<.01(1-tailed tests of significance)*

**Table G.3 CAAR estimated with Fama-French benchmark with equally weighted index over 300 days ending 30 days before the event (buyback subsample)**

| Day | Observations | CAAR | Positive: Negative | Portfolio Time Series CDA | CSec Err t | Generalized Sign Z |
| --- | --- | --- | --- | --- | --- | --- |
|  |  |  |  |  |  |  |
|  |  |  |  |  |  |  |
| (-30,-2) | 80 | -2.57% | 30:53 | -1.734** | -2.361** | -2.141* |
| (-1,0) | 80 | .59% | 44:39 | 1.508* | 1.315* | .935 |
| **(0, 0)** | **80** | **.77%** | **52:31** | **2.782***** | **2.164**** | **2.693***** |
| **(0,+1)** | **80** | **.86%** | **52:31** | **2.218***** | **2.075**** | **2.693***** |
| (0,+2) | 80 | .73% | 49:34 | 1.541* | 1.560* | 2.034** |
| (0,+3) | 80 | .82% | 47:36 | 1.480* | 1.637* | 1.594* |

**p<.1, **p<.05, ***p<.01 (1-tailed tests of significance)*

**Web Appendix H**

**Table H.1 Robustness checks with data winsorized and trimmed at 1% level: Refranchising vs buyback subsamples**

|  |  | Winsorized at 1% | | | | Trimmed at 1% | | | |
| --- | --- | --- | --- | --- | --- | --- | --- | --- | --- |
| **Dependent variable: Abnormal Stock Returns (0;+1), Market-adjusted Benchmark** | **Hypotheses** | Refranchising subsample (a) | | Buyback subsample (b) | | Refranchising subsample (a) | | Buyback subsample (b) | |
|  |  | Coef. | Robust Std. Err. | Coef. | Robust Std.Err. | Coef. | Robust Std. Err. | Coef. | Robust Std. Err. |
| Firm Royalty Rate | H2a(-), b(+) | -.043*** | .021 | .014*** | .006 | -.043*** | .018 | .017*** | .007 |
| Firm Advertising Intensity | H3a(-), b(+) | -.052 | .193 | .012 | .316 | -.038 | .174 | -.082 | .374 |
| Firm ROA | H4a(-), b(+) | -.131*** | .078 | .047 | .054 | -.113*** | .048 | -.054 | .065 |
| Firm Trade Credit Provided | H5a(+), b(-) | .065*** | .025 | -.003 | .023 | .070*** | .023 | .001 | .027 |
| Industry Dynamism | H6a(+), b(-) | .687 | .437 | .351 | .267 | .778** | .399 | .322 | .289 |
| Industry Munificence | H7a(-), b(+) | -.414*** | .171 | .239*** | .114 | -.261* | .147 | .240* | .131 |
| Controls |  |  |  |  |  |  |  |  |  |
| Firm Size |  | .003 | .003 | -.004 | .004 | .002 | .003 | -.004 | .004 |
| Free Cash Flow |  | .075*** | .040 | -.008 | .028 | .091*** | .034 | -.013 | .038 |
| Industry Concentration |  | .000 | .000 | .000 | .000 | .000 | .000 | .000 | .000 |
| Inverse Mills Ratio |  | -.002 | .003 | -.000 | .008 | .002 | .003 | -.004 | .009 |
| SIC5812 dummy |  | .077*** | .021 | .037 | .031 | .057*** | .023 | .035 | .034 |
| Year controls included in all specifications | | | | | | | | | |
| Intercept |  | -.026 | .024 | -.008 | .043 | -.049** | .024 | .010 | .040 |
| Observations | | 125 | | 80 | | 123 | | 78 | |
| Wald Chi2 | | 2890000*** | | 4780219.90*** | | 2348916.97*** | | 26552.23*** | |
| R sqr | | .401 | | .360 | | .347 | | .270 | |

**p<.1, **p<.05, ***p<.01 (2-tailed tests of significance)*

**Web Appendix I**

**Table I1. Main model results with inverse Mills ratio excluded**

| **Dependent variable: Abnormal Stock Returns (0; +1), Market-adjusted Benchmark** | **Hypotheses** | Refranchising subsample (a) | | Buyback subsample  (b) | |
| --- | --- | --- | --- | --- | --- |
|  |  |  |  |  | |
|  |  | Coef. | Robust Std. Err. | Coef. | Robust Std. Err. |
|  |  |  |  |  |  |
| Firm Royalty Rate | H2a(-), b(+) | -.041** | .019 | .016*** | .006 |
| Firm Advertising Intensity | H3a(-), b(+) | .060 | .255 | -.097 | .401 |
| Firm ROA | H4a(-), b(+) | -.163*** | .066 | -.058 | .065 |
| Firm Trade Credit Provided | H5a(+), b(-) | .068*** | .022 | .011 | .020 |
| Industry Dynamism | H6a(+), b(-) | .506 | .402 | .258 | .181 |
| Industry Munificence | H7a(-), b(+) | -.362*** | .156 | .208 | .131 |
| Controls |  |  |  |  |  |
| Firm Size (ln) |  | .004 | .003 | -.005 | .003 |
| Free Cash Flow |  | .095*** | .037 | .016 | .038 |
| Industry Concentration |  | .000 | .000 | .000 | .000 |
| SIC5812 dummy |  | .070*** | .021 | .030 | .026 |
| Year controls included in all specifications | | | | | |
| Intercept |  | -.031 | .024 | .009 | .043 |
| Observations | | 125 | | 80 | |
| Wald Chi2 | | 1828050*** | | 2007.98*** | |
| R square | | .407 | | .335 | |

**p<.1, **p<.05, ***p<.01 (2-tailed tests of significance)*

**References**

Hamilton, B. H., & Nickerson, J. A. (2003). Correcting for endogeneity in strategic management research. *Strategic organization*, *1*(1), 51-78.

Heckman, J. J. (1979). Sample selection bias as a specification error. *Econometrica: Journal of the Econometric Society*, 153-161.

Malshe, A., & Agarwal, M. K. (2015). From finance to marketing: The impact of financial leverage on customer satisfaction. *Journal of Marketing*, *79*(5), 21-38.

Shane, S. A. (1996). Hybrid organizational arrangements and their implications for firm growth and survival: A study of new franchisors. Academy of management journal, 39(1), 216-234.
